# An Improved Randomized Local Binary Features for Keypoints Recognition

**DOI:** 10.3390/s18061937

**Published:** 2018-06-14

**Authors:** Jinming Zhang, Zuren Feng, Jinpeng Zhang, Gang Li

**Affiliations:** 1 State Key Laboratory for Manufacturing Systems Engineering, Xi’an Jiaotong University, No. 28 Xianning West Road, Beilin District, Xi’an 710049, China; fzr9910@mail.xjtu.edu.cn (Z.F.); lg0993@stu.xjtu.edu.cn (G.L.); 2 Institute of Automation, Chinese Academy of Sciences, Beijing 100190, China; zhjphust@163.com

**Keywords:** binary feature, keypoints recognition, random ferns, random forests, ORB, SIFT

## Abstract

In this paper, we carry out researches on randomized local binary features. Randomized local binary features have been used in many methods like RandomForests, RandomFerns, BRIEF, ORB and AKAZE to matching keypoints. However, in those existing methods, the randomness of feature operators only reflects in sampling position. In this paper, we find the quality of the binary feature space can be greatly improved by increasing the randomness of the basic sampling operator. The key idea of our method is to use a Randomized Intensity Difference operator (we call it RID operator) as a basic sampling operator to observe image patches. The randomness of RID operators are reflected in five aspects: grids, position, aperture, weights and channels. Comparing with the traditional incompletely randomized binary features (we call them RIT features), a completely randomized sampling manner can generate higher quality binary feature space. The RID operator can be used on both gray and color images. We embed different kinds of RID operators into RandomFerns and RandomForests classifiers to test their recognition rate on both image and video datasets. The experiment results show the excellent quality of our feature method. We also propose the evaluation criteria for robustness and distinctiveness to observe the effects of randomization on binary feature space.

## 1. Introduction

Many computer vision tasks such as vision-based sensors [1], action recognition [2], image classification [3] ,visual detection [4], motion tracking [5], visual SLAM [6,7,8] and robot navigation [9] all rely on highly precise matching of local binary features extracted from different views of target images. There already exist many local invariant features such as SIFT [10], SURF [11], BRIEF [12], ORB [13] and AKAZE [14] that have achieved some success. However, these methods usually need careful preprocessing and complicated hand-designed sampling patterns to resist deformations like rotation, zooming and view-point changes. The other way to solve this problem is to treat keypoints matching as a classification problem, in which each class corresponds to the set of all possible views of such a point. RandomTrees classifier [15,16] and its variant [17], RandomFerns classifier [15,18] and restricted Boltzmann machine [19] are proposed to recognize keypoints. However, these classifier-based methods focus their attention on classifier improvement but ignore improving the quality of binary feature space. The basic sampling operator they used to construct feature space are nonrandom-weighted aperture-fixed two-grids single-channel intensity difference operators. Their randomness only reflects in the distribution of sampling position within an size-fixed image patch.

In this paper, we propose using more general randomized intensity difference sampling operator (we call it as RID sampling operator) to construct binary feature space for keypoints recognition. Comparing with the traditional randomized intensity tests sampling operators (we call it as RIT sampling operator) used in BRIEF [12], ORB [13], RandomizedTrees [16] and RandomFerns [18], Our RID sampling operators have much more randomness, which reflects in five aspects: the number of sampling grids within each operator, the distribution of operator position, the size of operator aperture, the weights of operator grids and the channel of operator grids. After randomly generating a specified number of RID sampling operators, we first divide them into a specified number of groups and then apply binary encoding method on each group to construct feature space. The main property of our completely randomized RID sampling operators is that they can provide multi-resolution observation and bring much more sampling redundancy than traditional incompletely randomized intensity difference sampling operators. Comparing with other non-randomized multi-resolution sampling patterns like BRISK [20] and FREAK [21], our RID operators need no special artificial design of sampling pattern and can be easily extended to process RGB (or other multi-channel) images. In order to observe the influences of operator aperture and weights on the quality of binary feature space, we take recognition rate as the objective function to select optimal aperture sizes for different kinds of RID sampling operators. We embed different kinds of RID sampling operators into RandomFerns classifier and RandomTrees classifier to test their recognition performance on both image dataset and video dataset. The experiment results indicate that our completely randomized RID sampling operators can obviously improve the quality of local binary feature space and indeed have excellent performance in keypoints recognition application. Another contribution of our work is that we propose evaluation criteria for robustness and distinctiveness of local binary features. The criteria are used to observe the effects of randomization on the quality of binary feature space. By observing the effects of aperture randomization and weighting randomization on the robustness and distinctiveness of the feature space, we reveal why the use of fully randomized basic sampling operators can effectively improve the separability of local binary features.

## 2. Related Works

Randomized local binary features have been widely used in keypoints matching and patches recognition. Their main characteristics are two points: one is randomly sampling within size-fixed image patch, the other is binary encoding of the sampling values. Many methods, including BRIEF [12], ORB [13] and AKAZE [14], extract a bits vector from keypoint neighborhood as its descriptor and matched by Hamming distance. Comparing with methods like SIFT [10], SURF [11] and KAZE, randomized local binary feature methods have many advantages, such as easy implementation, highly efficient computation and good enough matching precision. The basic sampling operator used in BRIEF, ORB and AKAZE are the same: the mean intensity difference of two rectangular grids randomly pre-selected within an image patch. However, the two small grids used for intensity binary comparison are with fixed aperture, fixed weight and fixed channel. There also exist some non-randomized local binary feature methods like BRISK and FREAK which always need a hand-designed specific sampling pattern. The disadvantage of descriptor-based methods is the requirement of rotation estimation. Their performances are largely depend on a good rotation estimation method. Classification-based keypoints recognition methods treat keypoints matching as a classification problem, in which each class corresponds to the set of all possible views of such a point [15]. These methods need no rotation estimation. Lepetit et al. [16] proposed using randomized trees as the classification technique. It is robust to illuminations changes, scale changes and occlusions. In their implementation, the tests performed at the tree nodes are simple binary tests based on the intensity difference of two sampling points with size-fixed blurring. Ozuysal et al. [18] showed that formulating the problem in a naive Bayesian classification framework makes many preprocessing operations unnecessary and produces an algorithm that is simple, efficient, and robust. To recognize the patches surrounding keypoints, the classifier uses hundreds of simple binary features and assumes independence between arbitrary sets of features. The basic sampling operators are also two size-fixed small grids pre-selected from keypoint neighborhood. S. Shimizu and H. Fujiyoshi [17] proposed using two-stage randomized trees for keypoints recognition. The viewpoints of the input image are classified in the first stage; in the second stage, keypoint classification is performed using the RTs trained with image viewpoints that are near those classified in the first stage. The features they used in tree nodes are also binary tests similar to [16]. Yuan et al. [19] proposed using RBM (Restricted Boltzmann Machine) classifier for keypoints recognition. The features set used to train an RBM model is defined by a n-dimensional binary vector based on the intensity difference of the two pre-selected pixels within image patches. These classification-based keypoints recognition methods differ from each other by the classifier they employed but use the same basic sampling operator. Wang et al. [3] proposed a new local neighborhood encoding method call random sampling LBP (RSLBP). RSLBP is different from the original local binary pattern (LBP) operator or LBP variants that adopt the difference between the neighboring pixels and the center pixel to describe the pixel. Based on the distribution of the image difference signal, point pairs are randomly selected in the local neighborhood, and LBP encoding is carried out after comparing the sums of pixels neighboring the random point. The basic sampling operator RSLBP employed is also pixel-pairs intensity tests, which is the same with [12,18].

After reviewing the existing randomized local binary feature methods, we find that the common ground of these methods is that they all use nonrandom-weighted aperture-fixed point-pairs single-channel basic sampling operators to construct binary feature space. However, our researches in this paper indicate that those incompletely randomized sampling operators are not conducive to the separability and diversity of binary feature space and can can not give full play to the performance of the local binary feature method.

## 3. Methods

### 3.1. Randomized Intensity Sampling Operators

In this subsection, we discuss the design of basic sampling operators. The traditional basic sampling operators used in [16,18,19] are usually nonrandom-weighted aperture-fixed position-randomized single-channel operators, which means they have size-fixed smoothing aperture and can be only applied on single channel images. Their randomness is only reflected by sampling positions. According to the notation in [12,16,18], we denote the type of traditional sampling operator as RIT (Randomized Intensity Tests) operator. One intensity test refers to randomly sampling two pixel points (or two smaller pixel areas) within image patch and comparing them with intensity, which is defined as follows [18]:

(1) $$\tau \left(p_{1},p_{2}\right)=\tau \left(p_{1}-p_{2},0\right)=\left\{\begin{array}{cc} 1 & p_{1}>p_{2}\\ 0 & p_{1}\leq p_{2} \end{array}\right.$$

The RIT operator is also used in the sampling patterns such as RSLBP [3], ORB [13], FREAK [21] and BRISK [20].

Differently, we use completely randomized RID (Randomized Intensity Difference) sampling operators to construct binary feature space. RID operator is random-weighted random-distributed multi-granular multi-channel sampling operator, which can be defined as follows:

(2) $$fv=op\left(patch\right)=\sum_{i=1}^{n}w_{i}*mI\left(cell_{i}\left(x_{i},y_{i},w_{i},h_{i},ch_{i}\right)\right)$$

where $cell_{i}$ is rectangular sampling area (a small pixel region) at randomly pre-selected position within image patch. *n* is the number of sampling cells within one RID operator. Each sampling cell $cell_{i}$ has its own position $\left(x_{i},y_{i}\right)$, aperture $\left(w_{i},h_{i}\right)$ and channel $\left(ch_{i}\right)$. $w_{i}$ is the weight of the $cell_{i}$. mI(·) represents the mean intensity operation on sampling cell, which ranges from 0 to 255 on 8-bits depth image. To ensure RID sampling operator to be gradient-like operator and the theoretical mean value of op(patch) equals zero, the weights of all sampling cells are set to meet the conditions as follows:

(3) $$\sum_{i=1}^{n^{+}}w_{i}^{+}=1\;\mathrm{and}\;\sum_{i=1}^{n^{-}}w_{i}^{-}=-1$$

where $w_{i}^{+}$ and $w_{i}^{-}$ represent the positive and negative weights within sampling operator, respectively. And $n^{+}+n^{-}=n$.

**Figure 1:**
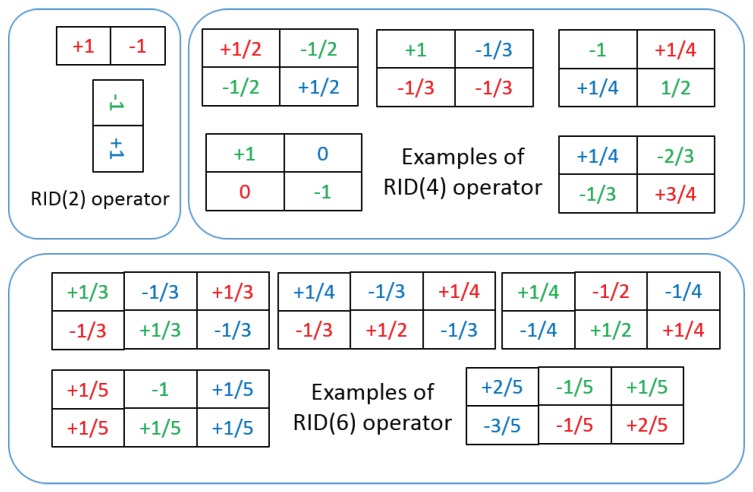
This figure shows some examples of three kinds of RID operators. The weight numbers of sampling cells within each operator are pre-selected randomly according to Equation (3) when generating operators. However, the weights of RID(2) operator have no randomness when image rotation occurring. The color of weight number of each cell is used to represent the sampling channel of that cell. Each operator can put its several cells into different channels so as to implement multi-channel joint randomized binary coding. These randomized sampling operators are pre-generated before applying them on image patches of all keypoints. Once generated, they will stay the same for all images.

Equations (2) and (3) define a general form of basic RID sampling operator. The number of sampling cells within each RID operator can be 2, 4, 6 or more. Therefore, we use RID(*n*) to represent the number of cells within a RID operator. For example, RID(4) means *n* = 4. As discussed in Section 3.2, in order to build *M*-dimensional feature space, we need pre-generate numbers of RID operators and arrange them into *M* groups. Within each group, the number of sampling cells among different RID operators can be different from each other. RID(2,4) means there are two kinds of RID operator in each group: RID(2) and RID(4). RID(2,4,6) means there are three kinds of RID operator in each group: RID(2), RID(4) and RID(6). The aperture size of RID operators within each group is also randomly pre-selected in a specified range. Because keypoint neighborhood is usually a 31 × 31 image patch, the aperture range of RID operator cannot be larger than 31 × 31. In our paper, RID(2)[6,20] means the aperture size of RID(2) operator is randomly selected in the range [6 × 6, 20 × 20]. RID(4)[3,30] means the aperture size of RID(4) operator is randomly selected in the range [3 × 3, 30 × 30]. The weights of sampling cells within each RID operator are also randomly generated according to the condition in Equation (3). According to the condition in Equation (3), RID(2) operator has only two cells and its weights have only two possible settings: [−1,+1] or [+1,−1]. Considering the rotation of image patches, the weights of RID(2) operator actually have no randomness. RID(4) operator has four sampling cells and its weights have many possible settings, like (1/2,1/2,−1/2,−1/2), (1/2,−1/2,1/2,−1/2), (1,−1/3,−1/3,−1/3), (−1,1/4,1/4,1/2), (−3/4,1/3,2/3,−1/4), and so on. We can see that under the constraint of condition in Equation (3), the weights of RID(4) operator have much more randomness than weights of RID(2) operator. As RID(6) operator has more sampling cells than RID(2) and RID(4), the weights of RID(6) operator also have many possible settings (see Figure 1) and have much more randomness than RID(2) and RID(4) operators. These multi-cell sampling operators have another advantage is that they can be easily extended to process rgb or other multi-channel images. In our methods, sampling cells of RID operator are randomly specified into different image channels when they are generated (see the rgb color labeled on weights number in Figure 1), which allows us to implement multi-channel joint randomized binary coding.

### 3.2. Binary Feature Space Construction

In this subsection, we discuss how to construct binary feature space using numbers of basic sampling operators.

We denote *M*-dimensional feature vector extracted from an image patch as: $V=\{v_{1},\cdots ,v_{m},\cdots ,v_{M}\}$. Let fv=op(patch) represent a general sampling operation within an image patch, where fv is sample value and op is a basic sampling operator. Each feature component $v_{m}$ corresponds to a group of basic sampling operators, denoted as $group_{m}=\left\{op_{1},\cdots ,op_{s},\cdots ,op_{S}\right\}$. In general, feature space construction is to design a mapping from $group_{m}$ to $v_{m}$, which can be expressed as $v_{m}=h\left(group_{m}\right)$. There are many possible approaches to design the mapping h(·).

The mapping h(·) used in local binary feature methods is to apply binary encoding operations on $group_{m}$, as follows:

(4) $$v_{m}=h\left(group_{m}\right)=\sum_{s=1}^{S}\left(2^{s-1}*\tau \left(op_{s},T\right)\right),\;op_{s}\in group_{m}$$

where τ(·,·) is a binary comparator defined as this: if $v_{1}>v_{2}$ then $\tau (v_{1},v_{2})$ = 1; otherwise $\tau (v_{1},v_{2})$ = 0. *T* is a threshold that always equals the theoretical average value of sampling operators in $group_{m}$. Since τ(·,·) is either 1 or 0, the value space of $v_{m}$ is a finite discrete integer set as follows:

(5) $$v_{m}\in F=\left\{0,1,2,\cdots ,2^{S}-1\right\}$$

Therefore, the value size of feature space *F* is controlled by the number of basic sampling operators in each group: $\left|F\right|=2^{S}$. The dimension of binary feature vector *V* is M∗S bits, because each feature component $v_{m}$ has *S* bits according to Equation (4) and we have *M* feature components. In the performance comparison experiments of Section 5.2 and Section 5.5, the parameter *S* is set to be 8 and *M* is set to be 40 for all the compared methods. Therefore, the dimension of binary feature vector is 320 bits (40 bytes) in those experiments.

If sampling operators are intensity-test or intensity-difference operators, the threshold *T* can be set to zero. If we put the RIT operator of Equation (1) into Equation (4), we can obtain the binary features embedded with RIT operator as follows:

(6) $$v_{m}=h\left(group_{m}\right)=\sum_{s=1}^{S}\left(2^{s-1}*\tau \left(p_{i}-p_{j},0\right)\right);\;here,\;op_{s}=p_{i}-p_{j}$$

The binary encoding method in Equation (6) has been used in many methods, such as BRIEF [12], RandomFerns [18], RandomTrees [16], RSLBP [3], ORB [13], FREAK [21] and BRISK [20]. The encoding manner they employed is to compare the value of intensity-test with mean value (T=0), which can be called “mean binary encoding”.

In this paper, we propose that the mapping *h* can also be the form of cyclic binary encoding, as follows:

(7) $$v_{m}=h\left(group_{m}\right)=\sum_{s=1}^{S}\left(2^{s-1}*\tau \left(op_{s},\;op_{(s+1)\%S}\right)\right),op_{s}\in group_{m}$$

The main differences between our improved method and the traditional method are the form of basic sampling operators and the binary encoding method. Next, we will discuss the difference between mean binary encoding and cyclic binary encoding.

**Figure 2:**
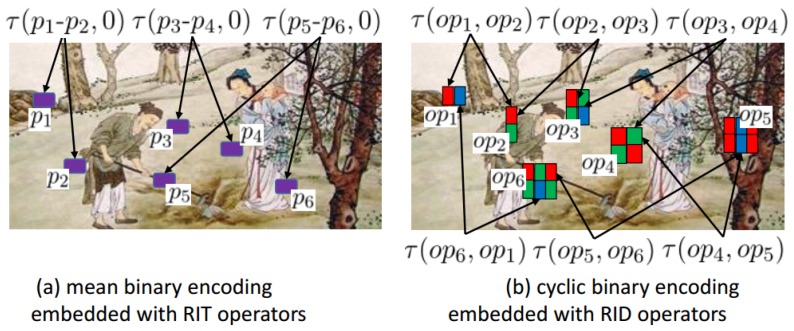
This figure shows the working process of binary encoding methods embedded with basic sampling operators. In the figure (**a**); each RIT operator just calculates the difference between two pixel regions: $op_{RIT}=P_{i}-P_{j}$. The theoretical average value of RIT operator equals 0. Therefore, the encoding manner of mean binary encoding method is to compare each $op_{RIT}$ with the average. In the figure (**b**); each RID operator calculates the difference of several pixel regions. The number of regions may be different between different RID operators. The rgb color of each sampling region represents the corresponding sampling channel. The theoretical average of RID operator also equals 0. However, cyclic binary encoding method does not compare RID sample value with 0. It compares one RID sample value with another RID sample value, which is better than that of comparing with theoretical average.

We explain the differences between the traditional binary feature methods and our improved feature methods in Figure 2. If we want to combine the traditional RIT operator with our proposed cyclic binary encoding method, the encoding Equation (6) can be rewritten as follows:

(8) $$v_{m}=h\left(group_{m}\right)=\sum_{s=1}^{S}\left(2^{s-1}*\tau \left(p_{i}-p_{j},p_{k}-p_{l}\right)\right);$$

**Figure 3:**
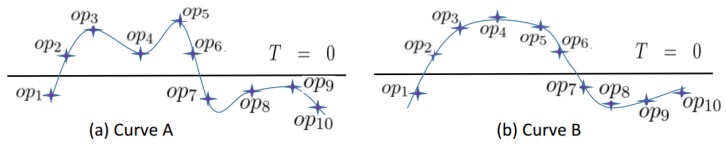
This figure is used to explain why cyclic binary encoding method (in Equation (7)) is better than mean binary encoding method (in Equation (4)). We sampled 10 times on two different curves (Curve A and Curve B) and got 10 sample values. Then we use different binary encoding methods to encode the sample values in order to compare the results of coding methods. Because the two curves are different from each other, we expect to obtain different binary-coding bits-string.

If we apply the mean binary encoding method of Equation (4) on two curves in Figure 3, we can get the bits-string like this:

$$\begin{array}{ccc} code-of-curveA & = & \tau \left(op_{1},0\right)\tau \left(op_{2},0\right)\cdots \tau \left(op_{9},0\right)\tau \left(op_{10},0\right)=0111110000\\ code-of-curveB & = & \tau \left(op_{1},0\right)\tau \left(op_{2},0\right)\cdots \tau \left(op_{9},0\right)\tau \left(op_{10},0\right)=0111110000 \end{array}$$

If we apply the cyclic binary encoding method of Equation (7) on two curves in Figure 3, we can get the bits-string like this:

$$\begin{array}{ccc} code-of-curveA & = & \tau \left(op_{1},op_{2}\right)\tau \left(op_{2},op_{3}\right)\cdots \tau \left(op_{9},op_{10}\right)\tau \left(op_{10},op_{1}\right)=0010110010\\ code-of-curveB & = & \tau \left(op_{1},op_{2}\right)\tau \left(op_{2},op_{3}\right)\cdots \tau \left(op_{9},op_{10}\right)\tau \left(op_{10},op_{1}\right)=0001111001 \end{array}$$

We can find that the binary codes obtained by applying Equation (4) are the same for both curves, but applying Equation (7) can obtain different binary codes for the two curves. Therefore, through the example of one dimensional curve’s binary encoding, we can see that cyclic binary encoding has greater ability to distinguish details than mean binary encoding. We find that the performance of Equation (7) is slightly better than the performance of Equation (4). Therefore, we employ the mapping in Equation (7) to construct feature space.

### 3.3. The Workflow of Our Methods

The workflow of our feature method has been given in Algorithm 1.

**Algorithm 1** The workflow of our RID feature extractor method**Input:** The number of groups for grouping all RID operators, *M*;The number of RID operators within each group, *S*;The number of sampling channels of RID operators, ch;Some keypoints detected from a given image, keypointsThe size of the image patch surrounding an keypoint, patch_sizeThe size range of RID operator aperture, aperture_range;**Output:** The feature descriptor set for the detected keypoints, descriptors;
  1:Randomly generating M×S RID sampling operators under the constraints of the parameters: ch, patch_size, aperture_range;  2:Randomly grouping M×S operators into *M* groups. Each group has *S* sampling operators;  3:**for** each keypoint∈keypoints **do**  4:    extracting the image patch surrounding the keypoint;  5:    applying the pre-generated and pre-grouped RID operators on the image patch to obtain sampling values by using Equation (2). Each operator return a intensity difference value to be negative or positive. As a result, these difference values are also grouped;  6:    **for** each group∈groups **do**  7:        applying the binary encoding method on the grouped sampling values to obtain the feature component value by using Equation (7);  8:    **end for**  9:    assembling all the component values into a bytes-string as the feature descriptor of this keypoint;  10:**end for**  11:Put the descriptor of each keypoint into the set: descriptors.  12:**return** descriptors;


## 4. Materials

**Figure 4:**
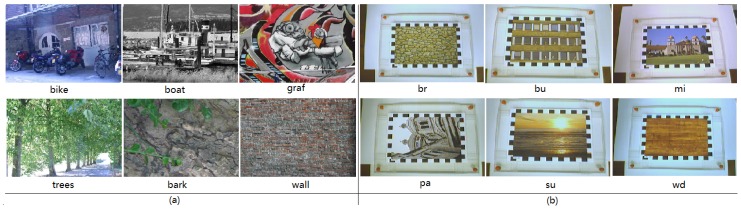
The datasets used in our experiments: (**a**) Wide baseline images set provided in the paper [22]; (**b**) Videos provided in the paper [5]. The videos are obtained through a controllable camera under situations of rotation, motion blur, lighting and so on. Both the two datasets, including all necessary material, are declared to be publicly available online and the download urls can be found in their papers.

We use three kinds of datasets to evaluate performances of different kinds of RID operators. The first dataset illustrated in Figure 4a is wide baseline images set selected from the dataset provided by Mikolajczyk et al. [22], which contains three structured images (bikes, boat, and graf) and three textural images (trees, wall, and bark). These images are used in the experiemnts of parameters selection in Section 5.1. The second dataset used in recognition rate experients for both RandomFerns and RandonmTrees classifiers embedded with different kinds of RID operators in Section 5.2 is VOC2011 dataset. The third dataset provided by Gauglitz et al. [5] consists of several videos obtained through a controllable camera under situations of rotation, motion blur, lighting and so on. Some video frame clips have been illustrated in Figure 4b. These videos are used by the planar object matching program in Section 5.5 to test matching and detecting performance under the complex and comprehensive continuous frame-to-frame deformations. We only select the six videos obtained under the situation of unconstrained camera trajectory. The six videos we selected are like this: “fi-xx-uc.avi”, where “xx” is the name of videos in Figure 4b. The ground-truth is frame-to-frame homography matrix file given by the author.

**Figure 5:**
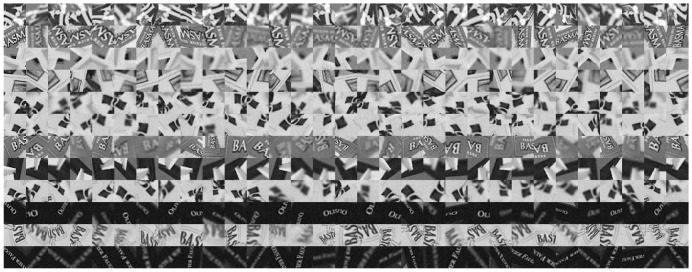
Image patch samples generated randomly for training and testing processes. Each row contains several possible appearances of a keypoint neighborhood.

We use multi-scale FAST detector to detect the specified number of keypoints on a reference image and assign a unique class id to each keypoint. The samples used to train and test classifiers are obtained by extracting patches surrounding the keypoint with size of 31 × 31 on randomly deformed images. Similar to random ferns [18], affine deformations can be expressed as 2 × 2 matrices, as follows:

(9) $$R_{\theta }R_{-\phi }diag\left(\lambda_{1},\lambda_{2}\right)R_{\phi }$$

where $diag(\lambda_{1},\lambda_{2})$ is a diagonal 2 × 2 matrix used as zooming factors and $R_{\gamma }$ represents a rotation of angle γ. Both to train and to test our classifiers, we warped the original images using such deformations computed by randomly choosing θ and ϕ in the [0:2π] range and $\lambda_{1},\lambda_{2}$ in the [0.6:1.5] range. We then add Gaussian noise with zero mean and a large variance 25 for gray levels ranging from 0 to 255 to these warped images. Some patch samples are illustrated in Figure 5, in which each row contains several possible appearances of a keypoint neighborhood. In the training procedure, we should let the classifier to see different appearances of a keypoint neighborhood as many as possible. So, we can tune the randomness and distorted degree of samples generating via these parameters to adapt the method to different application situations. For example, if we want applying the method to visual tracking based on keypoints matching, we can tune down or even close the rotation of image patch samples by setting θ and ϕ to be zero, because the tracking object may have no rotation during its motion. In the prediction procedure, we do not need the sample generation anymore. So these parameters have no effects on prediction.

## 5. Results

### 5.1. Parameters Selection for RID Operators

Sampling redundancy controlled by operator parameters has very important influence on the quality of feature space, so we will discuss the parameters selection in this subsection. When patch size is given, there are three important parameters that influence sampling redundancy: component count *M*, group size *S* and aperture range of RID operators. The dataset used in this subsection is illustrated in Figure 4a. In the experiments, we first detect 300 keypoints on each reference image and randomly generate 1000 image patch samples for each keypoint, then evaluate the recognition rate of RandomFerns classifier trained upon the feature space with specified parameter settings. The higher the recognition rate is, the parameter setting is better. The experiments are made on both gray images and color images.

#### 5.1.1. The Effects of the Number of Operators on Binary Feature Space

**Figure 6:**
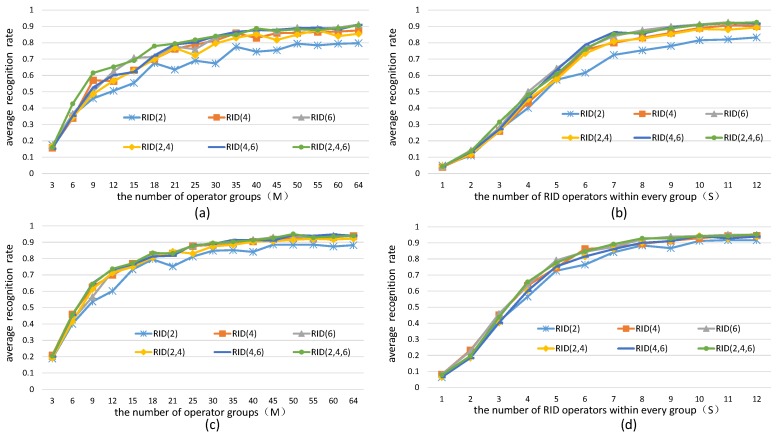
Average recognition rate curves of RandomFerns classifier embedded with different kinds of RID operators across all images in Figure 4a. The experiments in (**a**,**c**) are made by keeping *S* = 8 while increasing parameter *M*; the experiments in (**b**,**d**) are made by keeping *M* = 40 while increasing parameter *S*. The results in (**a**,**b**) are obtained from gray imags; (**c**,**d**) from color imags.

In this subsection, we keep aperture size of all sampling operators equal 15 × 15 and change parameters *M* and *S*, respectively. *M* is the number of feature components and *S* is the number of operators corresponded to each feature component. The experiment results are illustrated in Figure 6.

We can find from the results in Figure 6 that the recognition rate curves of RandomFerns classifier rise up rapidly and finally reach the saturation state along with the increasing of *M* and *S*. This means that increase the number of sampling operators can dramatically improve the quality of feature space, but still cannot eliminate the recognition error caused by the limitation of the classifier itself. In practice, the parameters *M* and *S* can not be too great to affects the computing efficiency. So, we chose *M* = 40 and *S* = 8 in our following experiments.

#### 5.1.2. The Effects of Operator Aperture on Binary Feature Space

**Figure 7:**
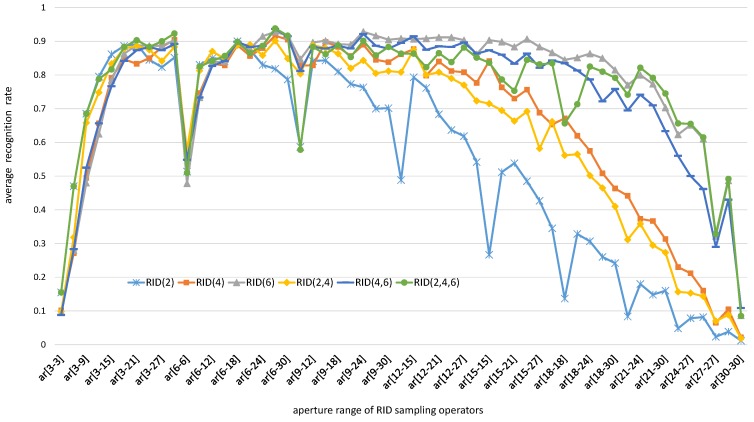
Each recognition rate curve is averaged across all images in Figure 4a. Each curve reflects the change of the recognition rate of RandomFerns classifier with the change of RID operator aperture. These experimental curves are obtained from gray images and the channels of sampling cells within each RID operator are the same.

**Figure 8:**
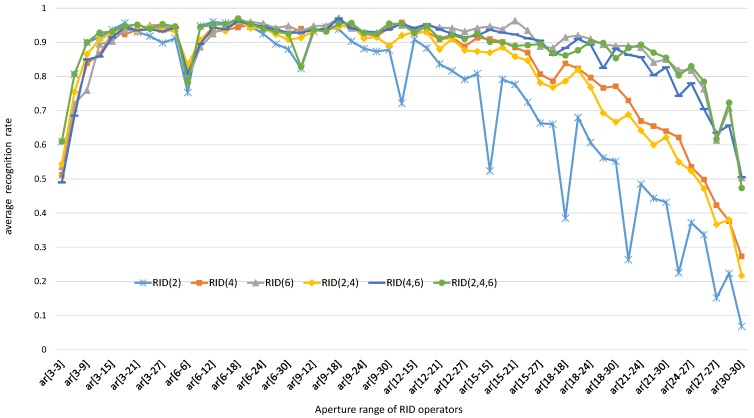
Each recognition rate curve is averaged across all images in Figure 4a. Each curve reflects the change of the recognition rate of RandomFerns classifier with the change of RID operator aperture. These experimental curves are obtained from rgb images and the channels of sampling cells within each RID operator are randomly specified when pre-generating RID operators.

In this subsection, we keep *M* = 40, *S* = 8 and change aperture size of RID operators to observe the influence of operator aperture on recognition rate of RandomFerns classifier. As the patch size is set to be 31 × 31, the aperture range of basic sampling operators can be from 3 × 3 to 30 × 30. In the experiments, we make operator aperture change in different ranges, like (3,6), (3,9), (3,12), ⋯. Under each aperture constraint, we test the recognition rate of RandomFerns classifier embedded with different RID operators. Finally, we plot the curves of recognition rate with respect to operator aperture range. The experiment results on both gray images and color images are illustrated in Figure 7 and Figure 8, respectively.

We can find from the experiment results in Figure 7 and Figure 8 that the recognition rate curves are significantly influenced by operator aperture size. When the parameters *M* and *S* are fixed, the larger the operator aperture is, the more sampling redundancy exits in feature space. When the operator aperture become too small or too large, the recognition rate curves of random ferns classifier become falling down in both gray images and color images. The reason behind this phenomenon is that large aperture sampling operators can resist noises but omit the details. On the contrary, small aperture sampling operators can capture details, but cannot resist noises. Therefore, sampling operator aperture should be selected randomly in a specified proper range. The best aperture range of the six kinds of RID sampling operators are different from each other, which can be due to the number of sampling cells is different in their sampling templates. According to the experiment results in Figure 7 and Figure 8, we can select a best aperture range for each kind of RID operators: ar[6,17] for RID(2) operator, ar[6,27] for RID(4), RID(6), RID(4,6) and RID(2,4,6) operators. In the following experiments, we will use these parameter settings to generate numbers of different types of RID operators.

#### 5.1.3. The Effects of Weights Randomization on Binary Feature Space

In this subsection, we make experiments to show the benefits of weights randomization to binary feature space. For comparison, we generate RID operators in two cases: in the first case the weights of sampling cells within every RID operator are non-randomized and kept fixed; in the second case the weights of sampling cells are randomly selected. In both cases the weights are set to meet the condition defined by Equation (3). Both random-weighted RID operators and nonrandom-weighted RID operators are embedded into RandomFerns classifier to observe their recognition rate with respect to operator aperture range.

**Figure 9:**
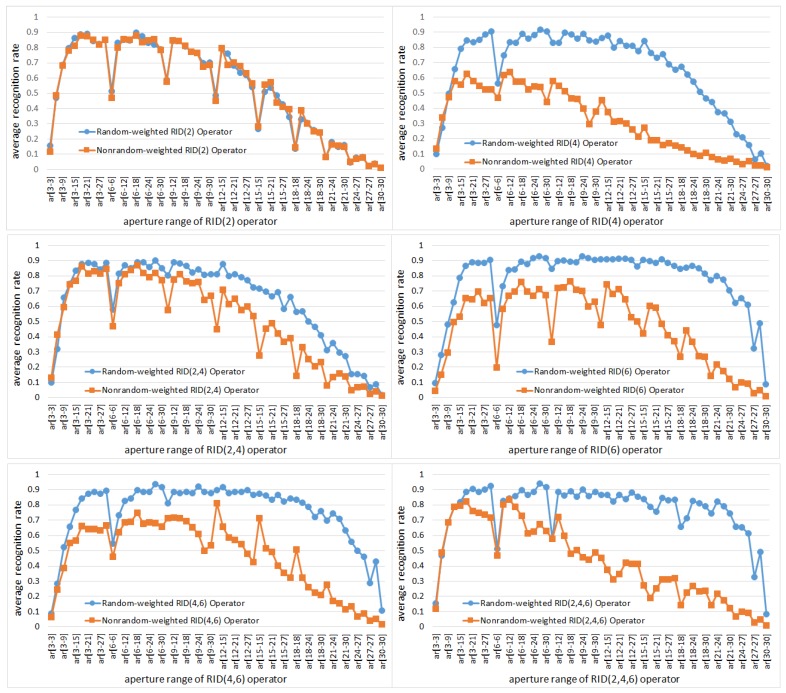
The comparison of recognition rate of random ferns classifier embedded with different RID operators. In each sub-figure two curves are plotted for comparison: one is for random-weighted RID operator, the other is for nonrandom-weighted RID operator. In both cases, recognition rate curves are plotted with respect to the range of operator aperture.

Figure 9 shows the experiment results, from which we can find three interesting phenomena. First, comparing the recognition rate curves of nonrandom-weighted RID operators and random-weighted RID operators in each sub-figure, we can find that random-weighted RID operators have much higher recognition rate than that of nonrandom-weighted RID operators. Further more, weight randomization makes the recognition rate curves look smooth, especially when operator aperture size become large. Second, comparing the recognition rate curves (blue curves) of different kinds of random-weighted RID operators, we find that the greater the weight randomness is, the less sensitive the recognition rate curve to the change of operator aperture. Because among the six kinds of RID operators, weight randomness of RID(6), RID(4,6) and RID(2,4,6) is greater than that of RID(2), RID(4) and RID(2,4). Another phenomenon deserved to be mentioned is the recognition rate curves of RID(2) operators. The two curves of random-weighted and nonrandom-weighted RID(2) operators are almost the same. The reason for this is that RID(2) operator has only two sampling cells and consequently has no weights randomness (See Figure 1). Similarly, the two curves in the subfigure of RID(2,4) operator are also close to each other, because RID(2,4) means there are RID(2) and RID(4) operators in each operator group and the number of RID(2) and RID(4) are randomly specified. The weights randomness of RID(2,4) is greater than RID(2) but less than RID(4).

### 5.2. Recognition Rate Tests on Images

In this subsection, we use different kinds of RID sampling operators to build binary feature space and combine the feature space with RandomFerns and RandomTrees classifiers [15] to test their recognition rate performance. We compare the performance between our cyclic binary encoding method embedded with RID sampling operators and the traditional mean binary encoding method embedded with RIT sampling operators. Traditional basic sampling operators are two-cells nonrandom-weighted aperture-fixed position-randomized operators, which are denoted as RIT(2)[ar,ar] in this paper, where ar can be set to be 3, 5, 7, 9, 11 or 13. So, the traditional binary feature methods are denoted like this: MBE-RIT(2)[ar,ar], where “MBE” refers to the traditional mean binary encoding method. While, our binary feature method is denoted as “CBE-RID($n_{1}$,$n_{2}$,$n_{3}$)[$ar_{1}$,$ar_{2}$]”, where “CBE” refers to the cyclic binary encoding method. “RID($n_{1}$,$n_{2}$,$n_{3}$)[$ar_{1}$,$ar_{2}$]” refers to the RID operator that has multiple random-distributed random-weighted sampling cells. The aperture size of RID operator is also randomly selected in the range ([$ar_{1}\times ar_{1}$],[$ar_{2}\times ar_{2}$]).

**Figure 10:**
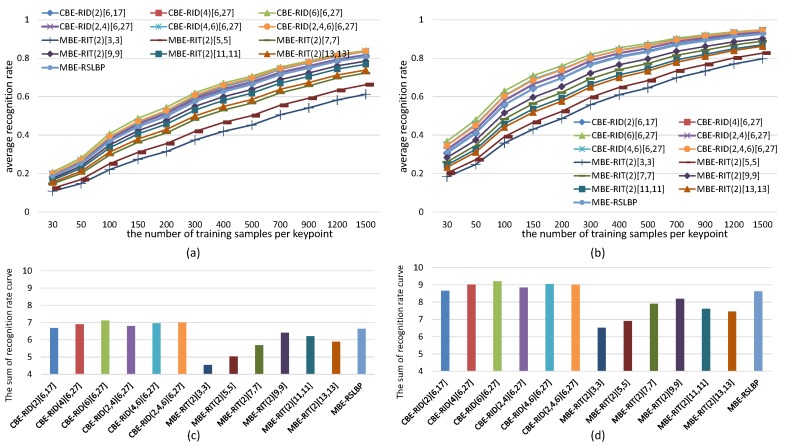
Average recognition rate curves of RandomTrees with respect to increasing training samples of per keypoint. Different curves represent the performance of different binary features. (**a**) Results obtained from gray images; (**b**) Results obtained from color images; Each bar of the histogram in (**c**) is calculated by accumulating the recognition rate curve of the corresponding feature method in (**a**); Similarly, (**d**) is calculated from (**b**). The histograms show the performance rank of compared methods.

**Figure 11:**
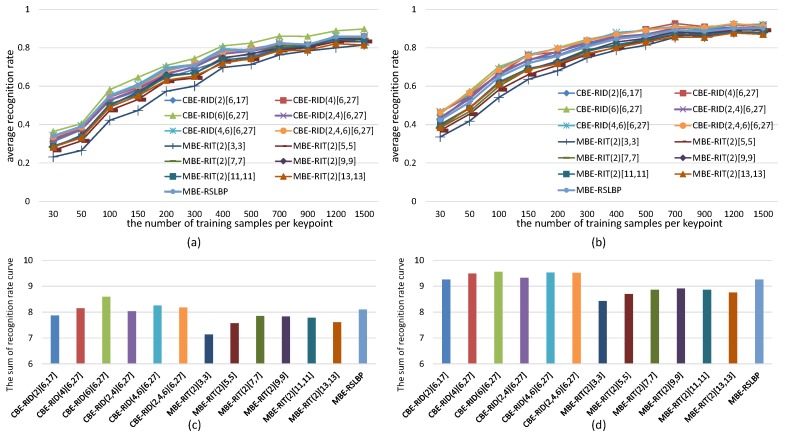
Average recognition rate curves of RandomFerns with respect to increasing training samples of per keypoint. (**a**) Results obtained from gray images; (**b**) Results obtained from color images; Each bar of the histogram in (**c**) is the AUC value of the corresponding feature method in (**a**); Similarly, (**d**) is calculated from (**b**). The histograms show the performance rank of these feature methods.

**Figure 12:**
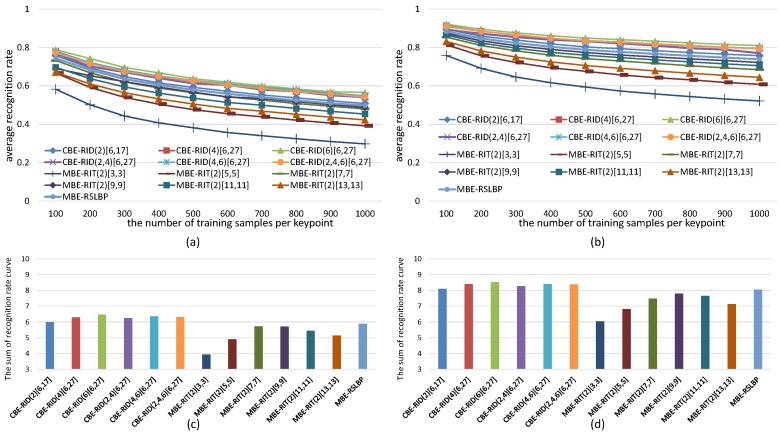
Average recognition rate curves of RandomTrees with respect to the growing number of keypoints on each reference image. Different curves represent the performance of different features. (**a**) Results obtained from gray images; (**b**) Results obtained from color images; Each bar of the histogram in (**c**) is the AUC value of recognition rate curve of the corresponding feature method in (**a**); Similarly, (**d**) is calculated from (**b**). The histograms show the performance rank of these feature methods.

**Figure 13:**
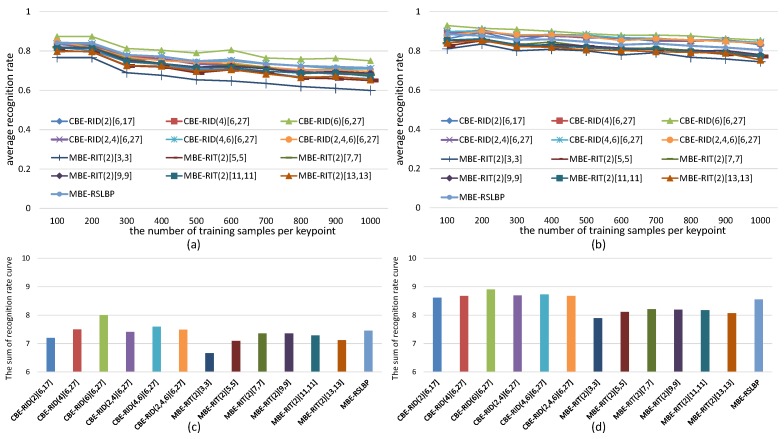
Average recognition rate curves of RandomFerns with respect to the growing number of keypoints on each reference image. (**a**) Results obtained from gray images; (**b**) Results obtained from color images; Each bar of the histogram in (**c**) is the AUC value of recognition rate curve of the corresponding feature method in (**a**); Similarly, (**d**) is calculated from (**b**). The histograms show the performance rank of these feature methods.

For the sake of fair, we set *M* = 40 and *S* = 8 within each method to keep the dimensions of feature space always same in the experiments. The experiments are made on both gray images and color images selected from VOC2011 dataset. The recognition rate of each tested method is computed in two cases: one test case is keeping the number of keypoints detected from each reference image fixed to be 300 while continuously increasing the number of training samples per keypoint, and the corresponding experiment results are illustrated in Figure 10 and Figure 11. The other test case is keeping the number of training samples per keypoint fixed to be 500 while increasing the number of keypoints on each reference image, and the corresponding experiment results are illustrated in Figure 12 and Figure 13. Because there are many compared feature methods and their performance curves are close to each other, it is hard to see which method is good. So, the AUC(area under curve) value of each feature method is computed by integrating the conresponding recognition rate curve. The AUC values of all methods are represented by a histogram so that we can see their performance differences and ranks at one glance.

The experimental results illustrated in Figure 10, Figure 11, Figure 12 and Figure 13 show four points: the first point is that the recognition rate performance of our completely randomized CBE-RID features is better than that of the traditional incompletely randomized MBE-RIT features in both RandomTrees and RandomFerns classifiers. This point indicates that complete randomization of the sampling operator can effectively improve the quality of the feature space regardless of what classifier you use. The second point is that the recognition rate performance of multi-channel randomized sampling operator is much better than that of single-channel sampling operator. Meanwhile, multi-channel random sampling and binary encoding does not bring a serious burden of calculation. We only need to calculate the integral images of multiple channels then randomly arrange numbers of sampling operators to different channel planes. In this way, we can establish a unified binary feature extraction method for color and gray images. The third point is that recognition rate performance rank of our completely randomized RID operators can be made from experimental results as this: RID(6)[6,27] ≥ RID(4,6)[6,27] ≥ RID(2,4,6)[6,27] ≥ RID(4)[6,27] ≥ RID(2,4)[6,27] ≥ RID(2)[6,17]. We can find from the performance rank that the operator with more sampling cells has better recognition rate. Our explanation for this phenomenon is that the operator with more sampling cells has more weights randomness and consequently has more diversity. Improving the diversity of the basic sampling operators is beneficial to the enhancement of the distinctiveness of the feature space. The last point we can find from the experimental results is that performance rank of the traditional incompletely randomized RIT operators is as this: RIT(2)[7,7] ≥ RIT(2)[9,9] > RIT(2)[11,11] ≥ RIT(2)[13,13] > RIT(2)[5,5] > RIT(2)[3,3]. This result is expected because the small aperture operators are easy to be disturbed by noise, which is not conducive to the robustness of the feature space, while the large aperture operators ignore many details, which is not conducive to the distinctiveness of the feature space. The randomization of sampling aperture can give consideration to both robustness and distinctiveness, which can effectively solve this problem (see the discussion in Section 6.3).

Now we discuss the computational burden of our method. To generate the sampling pattern, we only need to know the patch size of keypoint neighborhood, the aperture size range of sampling operator and the channels count of the image. This can be seen in algorithmic description in Section 3.1. The sampling pattern keep unchanged once they are generated randomly. We do not need to process the sampling pattern again like rotating and scaling the pattern. Our method is a kind of classifier-based keypoint recognition method, not the methods like ORB, BRISK and so on. The computation burden mainly happens on the classifier training procedure. In the training procedure, each keypoint is treated as a class and the original image patch of a keypoint will be randomly rotated and warped lots of times to make the classifier recognize as many as different views and scales of the image path. The sampling pattern stay the same for all training samples and test samples. Once the classifier is trained upon some keypoints detected from an image, it will be used to predict the image patch of an unknown-classified keypoint to one of the trained keypoints. In the prediction procedure, we also do not process any additional information about the sampling pattern generated. The prediction computation burden comes from two aspect: the first aspect is in the computation of integral image. If we apply our sampling operators on RGB images, we have to calculate the multi-channel integral images. The other aspect is in the image patch sampling process, which is very fast because of the use of integral images.

### 5.3. Comparison of Computational Efficiency

**Figure 14:**
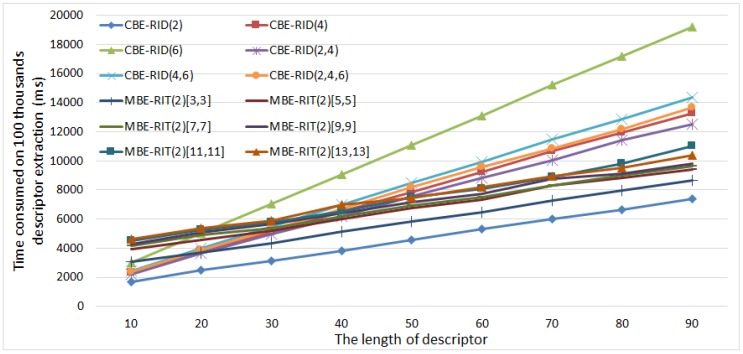
This figure illustrates the curves of consumed time of feature methods with respect to the increasing descriptor length. For each descriptor length, the test program is executed 100 loops and in each loop the feature method is applied on 100 thousands samples. The average consumed time across 100 loops is the total time that the feature method executes 100 thousand times.

In this subsection, we compare the computational efficiency between our CBE-RID features and traditional MBE-RIT features. In the comparison experiment, we first randomly generate 100,000 image patches from some given images, just as Figure 5 shows, then apply each kind of feature extractor method to the prepared patch samples to extract feature descriptors and record the consumed time. The test program contains 100 loops and within each loop feature descriptor extraction is executed on total 100,000 samples. Finally, we obtain the average consumed time for each feature method. The average time is the total time that the feature method executes 100,000 times. The comparison experiment is carried out on an Intel I7 CPU with 8 core inside. We only use one cpu core in the running. The experimental results are showed in Figure 14.

The results in Figure 14 show that the time costs of all feature methods are linear increasing with descriptor length. However, the linear increasing rates are different from each other. The traditional MBE-RIT feature methods have low linear growth rates, and the difference between MBE-RIT methods is not very large. While our CBE-RID feature methods have high linear growth rates, and the difference between CBE-RID methods is very large. We can find that the more cells we use in sampling operator (see the curve of CBE-RID(6)), the larger the linear growth rate of time consumption curve is. This is because the amount of floating-point calculation within one single operator is increased. However, When the descriptor length is less than 40, our CBE-RID methods (except CBE-RID(6)) consume less time than MBE-RIT methods. This is because our methods need no gaussian blurring on entire image patches. Therefore, our CBE-RID feature methods do not come with a significant calculation burden if the descriptor length is less than 50 bytes.

### 5.4. Comparison with the Existing State-of-Art Methods

In this subsection, we compare the matching precision performance between our CBE-RID feature method and the existing state-of-art feature methods under different test situations including rotation, zooming, viewpoint changing, blurring, noises and light changing. The existing methods selected to be compared are as follows: ORB, BRIEF, SIFT, SURF, KAZE, AKAZE, CSLBP, RILBP, RandomFerns and RandomTrees. Some of these methods have its own keypoint detector while some are only provide descriptor extractor. If a method has detector, we will use it; if not, we use the detector same as the author used in his original paper. The implementation of the methods we selected to compare are from OpenCV library. Our CBE-RID feature method is also a descriptor extractor method and the ORB detector is used in our programs. Therefore, our method is denoted as ORB-CBERID(6), which use one kind of RID variants: RID(6) operator.

**Figure 15:**
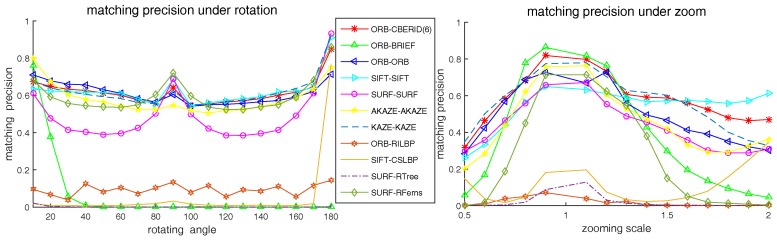
Matching precision results under image rotation and zooming.

**Figure 16:**
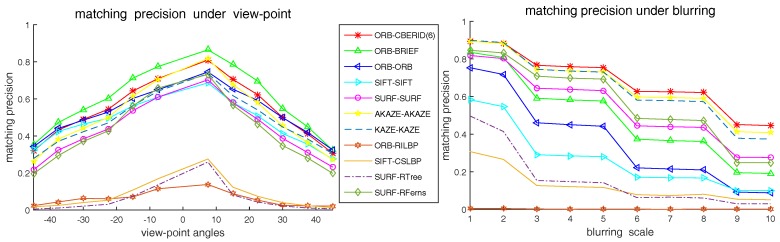
Matching precision results under image view-point and blurring.

**Figure 17:**
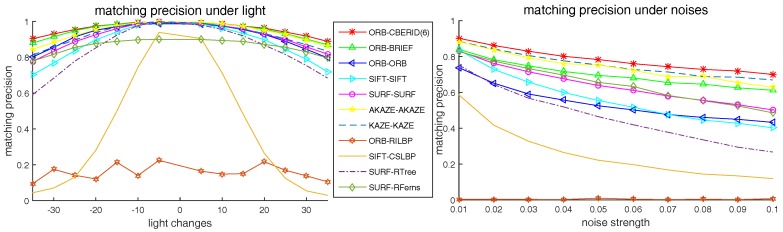
Matching precision results under image noise and lighting.

The experimental results are illustrated in Figure 15, Figure 16 and Figure 17. We can find from the experimental results that our CBE-RID feature method is not the best, but not bad, in the test of rotation, zooming and view-angle change. While in the test of blurring, noises and light changes, our CBE-RID feature method performs best. Overall speaking, our method performs well, which is competitive and can be comparable to those state-of-art methods.

### 5.5. Matching Precision Tests on Videos

**Figure 18:**
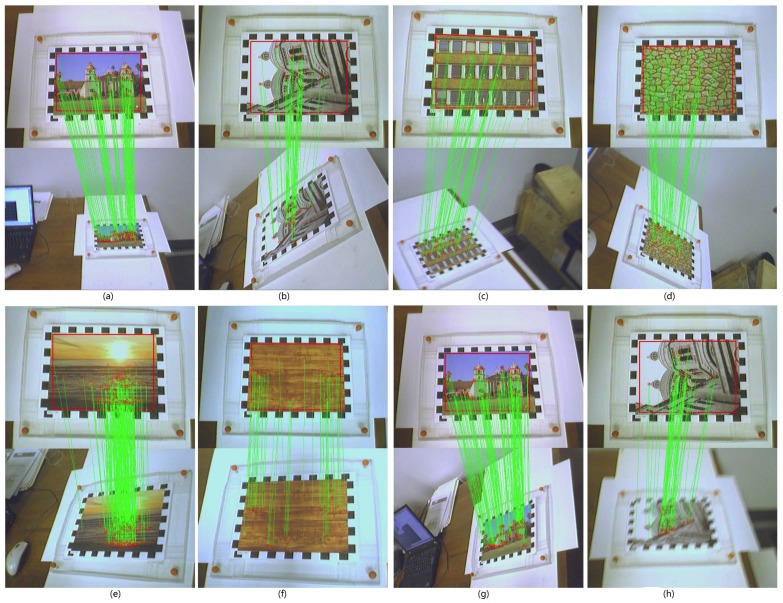
This figure shows the matching results on video frames. (**a**) is from video “mi”; (**b**) is from video “pa”; (**c**) is from video “bu”; (**d**) is from video “br”; (**e**) is from video “su”; (**f**) is from video “wd”; (**g**) is from video “mi”; (**h**) is from video “pa”. A variety of distortions, such as rotation, blurring, scaling, noises, and change of view, occur in motion. Matching precision is mainly influenced by two factors: one is the repeatability of keypoint detector, the other is the recognition power of keypoint descriptor. As we use the same keypoint detector (Multi-scale FAST detector) to provide keypoints for all descriptor methods, the matching results can reflect the recognition ability of each feature extraction methods.

In this subsection, we evaluate CBE-RID features in the real-world application of frame-to-frame matching on video dataset illustrated in Figure 4b. Each video contains a planar object undergoing motions involving a large range of rotations, blurring, scaling, and perspective deformations. We use a reference image in which the planar object is seen frontally to detect keypoints and train RandomFerns classifier model for the reference keypoint classes. The keypoints extracted from each input frame are then matched against those reference keypoints using RandomFerns classifier. Given keypoint matches between reference frame and input frame, we use the RANSAC method to estimate homography matrix then take all matches with re-projection error less than 5 pixels to be inlier matches. The number of keypoints detected on frames can be controlled by detector threshold. We use a low enough threshold to initially detect a large number of keypoints then retain strongest keypoints if the initial keypoint count on reference image is greater than 200. The number of keypoints on input frames varies from hundreds to thousands which largely depends on the object motions. For each kind of feature method, we set *M* = 40 and *S* = 8. Figure 18 shows some matching results on video frame clips.

**Figure 19:**
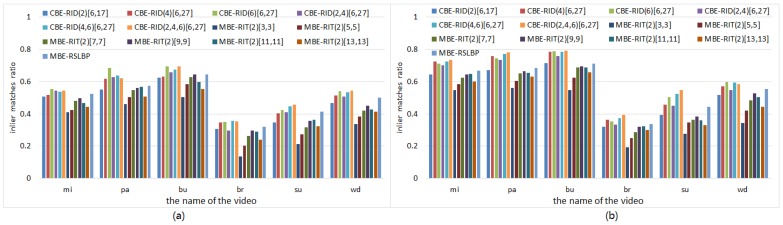
Moving planar object matching results: (**a**) is obtained from gray frames (**b**) is obtained from color frames.

The experiment results are illustrated in Figure 19, which represent the average inlier matches ratio across all frames of each video. Inlier matches ratio is computed from the proportion between the number of correct matches and total matches, which evaluates the matching precision performance. The experiment results in Figure 19 keep consistent with the evaluation results in Section 5.2 and further shows that our completely randomized CBE-RID features are better than the traditional incompletely randomized MBE-RIT features.

## 6. Discussion

In this section, we first give the evaluation criteria for robustness and distinctiveness of local features, then make experiments using our evaluation criteria to obtain an insightful observation and explanation for our completely randomized local binary feature method. In this section, we try to answer three questions: the first question is how to measure robustness and distinctiveness; the second question is that how the aperture size of basic sampling operators influences robustness and distinctiveness; The third question is that how the weights randomization of basic sampling operators influences robustness and distinctiveness.

**Figure 20:**
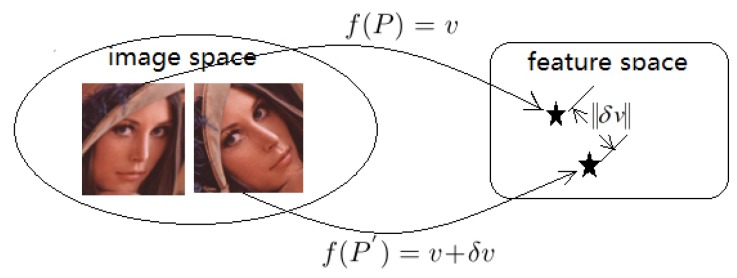
Feature method as a mapping from image space to feature space.

Local feature method can be considered as a mapping from image space to a special feature space, expressed as: f(P)=v, where *f* is a local feature method, *P* is an image patch, and *v* is descriptor vector of image patch *P*. Considering deformations occurring on image patch *P* as some kinds of disturbance, we have a mapping like this: $f(P^{{}^{\prime }})=v+\delta v$. The two mappings are illustrated in Figure 20.

### 6.1. How to Measure Robustness

**Figure 21:**
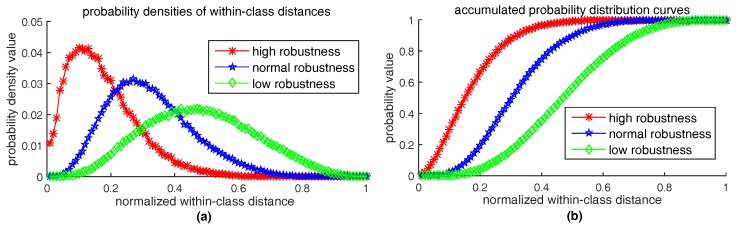
Three typical probability distributions of within-class distances obtained by RID(2,4,6): (**a**) is probability density curves; (**b**) is accumulated distribution curves.

The robustness of a feature method requires that feature mapping *f* has the ability of keeping invariant to some kinds of disturbance, which can be expressed as follows:

(10) $$∥f\left(P\right)-f\left(P^{{}^{\prime }}\right)∥=∥v_{P}-v_{P^{{}^{\prime }}}{∥=∥\delta v∥}_{norm}\leq \varepsilon_{r},\forall P^{\prime }$$

where $\varepsilon_{r}>0$ is a given positive number, and ${∥\delta v∥}_{norm}$ is the distance between the descriptor from original patch *P* and the descriptor from distorted patch $P^{\prime }$. The type of norm is determined by the type of feature space, for example, Hamming distance for bits-type vector or Euclidean distance for float-type vector. The smaller the upper bound $\varepsilon_{r}$ is, the more robust the feature method is. Although the upper bound $\varepsilon_{r}$ reflects robustness, using $\varepsilon_{r}$ as the robustness criterion is not appropriate, because $\varepsilon_{r}$ is a non-normalized value and the criterion should be relative value rather than absolute value. If we treat all descriptors extracted from possible appearances of a keypoint as a class, distances ∥δv∥ between the descriptors can be considered as within-class distances. Within-class distances reflect the clustering density of descriptor vectors in their feature space. The more denser the descriptor vectors distribute in their feature space, the more robust the feature method is. So, we propose an approach to observe and measure the clustering density of descriptors. Our approach is to calculate the probability distribution of within-class distances. Given a set of descriptors extracted from possible appearances of a keypoint, we first compute the distances between each other and normalize them into the range [0,1], then count the density histogram of the normalized distances. Three kinds of typical probability density curves of within-class distances are illustrated in Figure 21a. Their corresponding accumulated probability distribution curves (see Figure 21b) are obtained by integrating the probability density curves, as follows:

(11) $$F\left(d\right)=\sum_{d_{i}\leq d}p\left(d_{i}\right)$$

where variable *d* is normalized descriptor distance from 0 to 1. We use 100 bins in the density histogram and $p(d_{i})$ is the density value of *i*th bin. Figure 21 shows that the more robust the feature method is, the nearer the probability density curve is to vertical axis and thus the more rapidly the accumulated probability distribution curve rises up. Therefore, we can use the integration value of accumulated distribution curve of within-class distances to quantify robustness, as follows:

(12) $$robustness-score=\frac{1}{100}\sum_{d_{i}\leq 1}F\left(d_{i}\right);$$

∀i=1,2,⋯,100, $0\leq F(d_{i})\leq 1$. So, robustness-score ∈ [0,1] measures the area below the accumulated distribution curve of within-class distances.

### 6.2. How to Measure Distinctiveness

**Figure 22:**
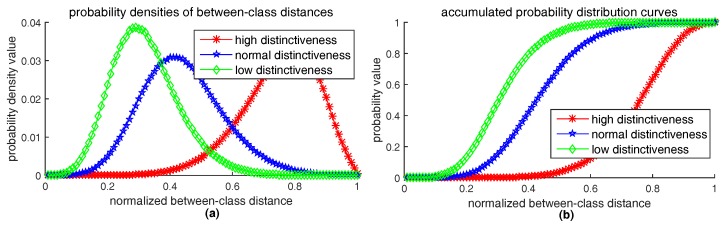
Three typical probability distribution curves of between-class distances obtained by RID(2,4,6): (**a**) is probability density curves; (**b**) is accumulated distribution curves.

The distinctiveness of a feature method requires that the feature mapping *f* can make descriptor vectors from different keypoints separable as much as possible in their feature space. Let *P* and *Q* be image patches of two different keypoints, distinctiveness can be expressed as follows:

(13) $$∥f\left(P\right)-f\left(Q\right)∥=∥v_{P}-v_{Q}{∥=∥\delta v∥}_{norm}\geq \varepsilon_{d},\;\forall Q\neq P$$

where $\varepsilon_{d}>0$ is a given positive number, $v_{P},v_{Q}$ is the descriptors extracted from image patch *P* and *Q*, respectively. The greater the lower bound $\varepsilon_{d}$ is, the more distinctive the feature method is. Since $\varepsilon_{d}$ is a non-normalized absolute value rather than a relative value, taking $\varepsilon_{d}$ as distinctiveness criterion is not proper. Let descriptors extracted from different keypoints belong to different classes, distances ∥δv∥ can be considered as between-class distances that measure the separable level of descriptor vectors from different classes. Similar to the situation of robustness evaluation, the separable level of descriptor vectors from different classes can be observed and measured by the probability distribution of between-class distances. Given two sets of descriptors extracted from two different keypoints, we first compute the between-class distances and normalize them into the range [0,1], then calculate the density histogram of the normalized distances. Three kinds of typical probability distribution curves of between-class distances are illustrated in Figure 22, which explains that the more distinctive the feature method is, the further the probability density curve is to vertical axis and thus the more slowly the accumulated distribution curve rises up. Therefore, we can use the integration value of accumulated distribution curve of between-class distances to quantify distinctiveness, as follows:

(14) $$distinctiveness-score=1-\frac{1}{100}\sum_{d_{i}\leq 1}F\left(d_{i}\right);$$

Similar to robustness-score in Equation (12), distinctiveness-score also ranges from 0 to 1, which measures the area above the accumulated distribution curve of between-class distances.

### 6.3. The Effects of Operator Aperture on Robustness and Distinctiveness

In this subsection, we discuss the effects of operator aperture on robustness and distinctiveness. To remove the influence of weights randomization and only consider the effect of the operator aperture, we use the nonrandom-weighted sampling operator to carry out the experiment. The robustness-score and distinctiveness-score are computed according to Equations (12) and (14), respectively.

**Figure 23:**
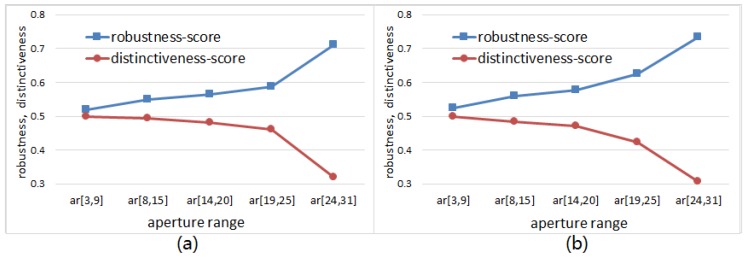
The curves of robustness-score and distinctiveness-score with respect to the aperture size range of nonrandom-weighted RID operator: (**a**) Curves obtained by using the nonrandom-weighted RID(4) operator; (**b**) Curves obtained by using the nonrandom-weighted RID(2,4) operator.

We can see from the experimental results illustrated in Figure 23 that the robustness-score keeps increasing with the growth of operator aperture; meanwhile the distinctiveness-score keeps decreasing with the growth of operator aperture. The experimental results here are in agreement with the experimental results in Figure 7. When using the same classifier, the recognition rate is determined by separability of feature space. While the separability is co-determined by robustness and distinctiveness. Unfortunately, Figure 23 shows us that we cannot improve robustness and distinctiveness at the same time. So, the RID operator aperture is randomly pre-selected in a larger range to trade-off between robustness and distinctiveness. The trade-off design skills are also used in BRISK [20] and FREAK [21]. Unlike their manual multi-resolution sampling pattern, we use the recognition rate as an evaluation criterion to select the most reasonable range of the operator aperture. This optimization process adopts a method similar to grid search to find good enough operator aperture distribution, then output a curve (see Figure 7) of the recognition rate with respect to the variation of aperture range. Our design is a statistical result from a large number of test data, rather than relying on human inspiration or intuition.

### 6.4. The Effects of Weights Randomization on Robustness and Distinctiveness

In this subsection, we discuss the effects of weights randomization on robustness and distinctiveness. We use the random-weighted sampling operator to carry out the experiment to compare with the results in Figure 23. The robustness-score and distinctiveness-score are computed according to Equations (12) and (14), respectively.

**Figure 24:**
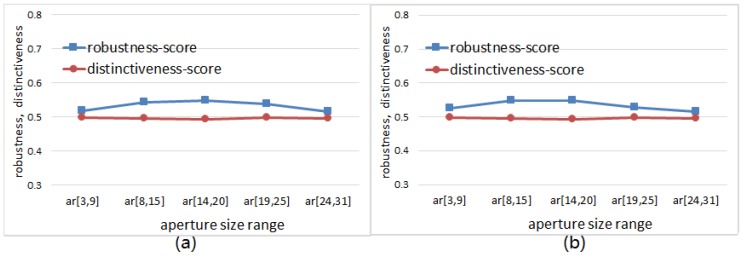
The curves of robustness-score and distinctiveness-score with respect to the aperture size range of random-weighted RID operator: (**a**) Curves obtained by using the random-weighted RID(4) operator; (**b**) Curves obtained by using the random-weighted RID(2,4) operator.

We can see from the experimental results illustrated in Figure 24 that the robustness-score keeps increasing first and then decreasing with the growth of operator aperture; meanwhile the distinctiveness-score keeps unchanged with the growth of operator aperture. The results here are distinctly different from the results in Figure 23. Due to the effect of random weighting, the distinctiveness of the feature space has not changed obviously when the aperture of the sampling operator becomes larger. Therefore, the random weighting improves the diversity of the basic sampling operators so that we do not lose the distinctiveness when we increase the robustness. However, we can also find in Figure 24 that the robustness-score is reduced when the operator aperture becomes particularly large. This phenomenon can just explain why the recognition rate curves of Figure 9 drop sharply when the operator aperture becomes particularly large. The experimental results in Figure 23 and Figure 24 indicate that the separability of binary feature space is comprehensively determined by robustness and distinctiveness.

## 7. Conclusions

In this paper, we carry out researches on randomized local binary features under the background of keypoints recognition and image patches classification. We make experiments to analyze several key parameters that have significant impacts on the quality of the binary feature space. The parameters related to basic sampling operator mainly include position, aperture, weights, channel, pattern and count, which can control the sampling redundancy and multi-resolution observation of local image areas. Through our experiments, we find that the binary feature space constructed by completely randomized basic sampling operator has very good quality compared with the traditional incomplete randomized basic sampling operator. Further, the improvement of the quality of the binary feature space by the complete randomization of the sampling operator is consistent regardless of what classifier you use. It is worth mentioning that this complete randomization does not bring more computational burden and can be easily implemented without need of elaborate manual design.

## Abbreviations

The following abbreviations are used in this paper:

BRIEF	Binary Robust Independent Elementary Features
ORB	Oriented FAST and Rotated BRIEF
AKAZE	Accelerated KAZE features
SIFT	Scale Invariant Feature Transorm
